# Krüppel-Like Factors in Vascular Inflammation: Mechanistic Insights and Therapeutic Potential

**DOI:** 10.3389/fcvm.2018.00006

**Published:** 2018-02-05

**Authors:** David R. Sweet, Liyan Fan, Paishiun N. Hsieh, Mukesh K. Jain

**Affiliations:** ^1^Case Cardiovascular Research Institute, Case Western Reserve University, Harrington Heart and Vascular Institute, University Hospitals Cleveland Medical Center, Cleveland, OH, United States; ^2^Department of Pathology, Case Western Reserve University, Cleveland, OH, United States

**Keywords:** Krüppel-like factor, Krüppel-like transcription factors, vascular inflammation, atherosclerosis, endothelial cells, vascular smooth muscle cells, macrophages

## Abstract

The role of inflammation in vascular disease is well recognized, involving dysregulation of both circulating immune cells as well as the cells of the vessel wall itself. Unrestrained vascular inflammation leads to pathological remodeling that eventually contributes to atherothrombotic disease and its associated sequelae (e.g., myocardial/cerebral infarction, embolism, and critical limb ischemia). Signaling events during vascular inflammation orchestrate widespread transcriptional programs that affect the functions of vascular and circulating inflammatory cells. The Krüppel-like factors (KLFs) are a family of transcription factors central in regulating vascular biology in states of homeostasis and disease. Given their abundance and diversity of function in cells associated with vascular inflammation, understanding the transcriptional networks regulated by KLFs will further our understanding of the pathogenesis underlying several pervasive health concerns (e.g., atherosclerosis, stroke, etc.) and consequently inform the treatment of cardiovascular disease. Within this review, we will discuss the role of KLFs in coordinating protective and deleterious responses during vascular inflammation, while addressing the potential targeting of these critical transcription factors in future therapies.

## Introduction

The role of inflammation in vascular disease is well recognized, involving dysregulation of both circulating immune cells as well as the cells of the vessel wall itself. Upon exposure to noxious stimuli (altered hemodynamics, circulating inflammatory factors, and oxygenation level), the vessel wall undergoes characteristic changes such as endothelial cell (EC) activation and vascular smooth muscle cell (VSMC) proliferation and migration, leading to the presentation of a “sticky” surface attractive to circulating monocytes and other immune cells. In certain contexts (e.g., acute thrombotic occlusion), this inflammation results in beneficial vascular remodeling that maintains proper perfusion to ischemic organs. During chronic insults, such as in dyslipidemia, unrestrained vascular inflammation leads to pathological remodeling that eventually contributes to atherothrombotic disease and its associated sequelae (e.g., myocardial/cerebral infarction, embolism, and critical limb ischemia).

Signaling events during vascular inflammation orchestrate widespread transcriptional programs that affect the functions of ECs, VSMCs, and circulating inflammatory cells. Central to many of these programs is the nuclear factor (NF)-κB signaling cascade. Upon stimulation by inflammatory stimuli [cytokines, oxidized low-density lipoprotein (oxLDL), glucose], regulatory cytosolic protein IκB is phosphorylated and targeted for ubiquitin–proteosome degradation. This liberates the normally sequestered cofactors p65 and p50 to translocate into the nucleus, where they mediate pro-inflammatory transcription [reviewed in Ref. (1)]. In addition, the coactivator p300 can complex with p65/p50 to stabilize the chromatin structure for effective transcription (2). Outside of NF-κB signaling, regulation of vascular inflammation can also occur through microRNAs (miRs). miRs are non-coding RNAs that regulate post-transcriptional gene expression by inhibiting mRNA translation. Within the scope of vascular inflammation, miRs can either enhance or diminish pathological inflammation, depending on the target genes [reviewed in Ref. (3)]. Multiple facets of vascular cell biology, including NF-κB and miR signaling, are regulated by Krüppel-like factors (KLFs). Within this review, we will discuss the role of KLFs in coordinating protective and deleterious responses during vascular inflammation, while addressing the potential targeting of these critical transcription factors in future therapies.

## KLFs Background

Originally discovered as homologs to the *Drosophila melanogaster* gene, Krüppel (4), KLFs belong to a family of zinc-finger containing transcription factors with roles in cellular development, differentiation, metabolism, and activation. There are 18 currently predicted mammalian KLFs expressed in various tissues and during periods of development. KLFs share within their C-terminal regions three highly conserved zinc-fingers recognizing a 5′-C(A/T)CCC-3′ consensus sequence often near target genes, though the sequence can occur in distant regions as well such as in enhancers. The amino-terminus functions in transactivation or repression and participates also in protein–protein interactions (5). For many KLFs, there is considerable overlap in gene targets within a single cell type. However, despite the homology of structure, binding sequences, and protein interaction targets, there are also substantial differences in downstream transcriptional effects between KLFs. Several excellent reviews are available discussing sequence homology, chromosomal location, and expression pattern of the KLFs (5, 6). Given their abundance and diversity of function in cells associated with vascular inflammation, understanding the transcriptional networks regulated by KLFs will further our understanding of the pathogenesis underlying several pervasive health concerns (e.g., atherosclerosis, stroke, etc.) and consequently inform the treatment of cardiovascular disease.

## EC KLFs

**Figure 1:**
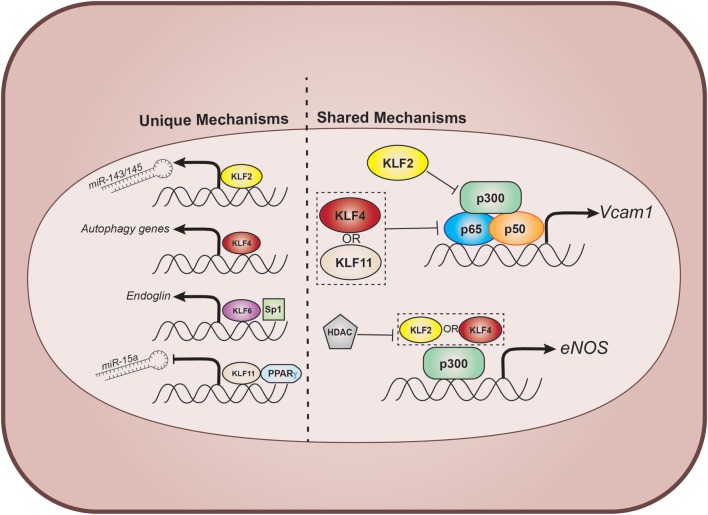
Select effector functions of endothelial KLFs. Within ECs, there are both unique and shared gene targets and binding partners for various KLFs. In general, KLF2, 4, and 11 resist endothelial adhesive transcription by binding to, and inhibiting, multiple cofactors of nuclear factor-κB signaling. Additionally, KLF2 and 4 promote transcription of the vasoprotective factor, eNOS, *via* cooperation with p300. This process is inhibited by recruitment of HDACs to the eNOS promoter. KLFs also affect endothelial function during vascular inflammation through unique transcriptional events that include modulation of miRs and stimulating protective cellular component recycling through autophagy. KLF, Krüppel-like factor; EC, endothelial cell; HDAC, histone deacetylase; VCAM-1, vascular cell adhesion molecule-1; eNOS, endothelial nitric oxide synthase; miR, microRNA; Sp1, specificity protein 1; PPARγ, peroxisome proliferator-activated receptor gamma.

The vascular endothelium acts as an initial sensor and transducer of inflammatory stimuli such as disturbed blood flow, cytokines, oxLDL, and advanced glycation end products, often responding with activation of classical inflammatory cascades which have been elegantly dissected over the past few decades. While brief bouts of inflammation, particular during wound healing, are an appropriate physiologic response, endothelial dysfunction resulting in sustained, chronic inflammation is central to a diverse array of cardiovascular diseases. The transcriptional regulation of endothelial inflammation therefore remains of critical interest. An accumulating body of evidence now exists defining key roles for several KLF transcription factors in the control of vascular inflammation, which we review below (Figure 1).

### Krüppel-Like Factor 2

Endothelial KLF2 has primarily anti-inflammatory, anti-thrombotic, and anti-migratory functions. As a regulator of inflammation, KLF2 inhibits both the expression of inflammatory cytokines and the production of adhesion molecules that are critical for leukocyte recruitment and extravasation (7–10). Through its binding to the transcriptional coactivator p300/CBP, KLF2 is capable of preventing NF-κB/p300 interaction and subsequent activation of the vascular cell adhesion molecule-1 (VCAM-1) promoter (11) Furthermore, the KLF2-p300 interaction permits KLF2 binding to the endothelial nitric oxide synthase (eNOS) promoter to induce transcription of this vasoprotective enzyme (11). These early studies demonstrated KLF2’s ability to influence transcription through direct DNA-binding and or indirect cofactor sequestration mechanisms. In addition to affecting the NF-κB pathway, studies have shown that KLF2’s anti-inflammatory effects are also produced through preventing the nuclear translocation of the inflammatory transcription factor ATF2 (9). In response to various stressors, JNK signaling leads to ATF2 nuclear translocation and successive inflammatory transcription. During shear stress, however, KLF2 induces cytoskeletal remodeling that eventually prevents JNK activation, and thus ATF2 translocation (12). Additionally, KLF2 provides protection against oxidative damage in ECs *via* the induction of hemeoxygenase-1 (13). Furthermore, ECs overexpressing KLF2 secrete atheroprotective miRs-143/145 in microvesicles that reduce atherosclerosis by targeting genes critical for VSMC dedifferentiation (*Mmp3, Elk1, Camk2d*) (14).

Thrombosis associated with atherosclerotic lesions contributes to many of the complications associated with atherosclerosis. Similar to its ability to repress endothelial inflammation, KLF2 modulates anti-thrombotic transcription. KLF2 binds directly to the promoter of thombomodulin-1, thereby increasing transcription of this potent anti-thrombotic and anti-inflammatory factor (8, 15, 16). Additionally, KLF2 inhibits the effects of thrombin-mediated endothelial activation by preventing transcription of thrombin’s receptor, PAR-1 (17). *In vivo*, there is a clear association between KLF2 levels and vascular inflammatory disease. While complete knockout of KLF2 is embryonically lethal (18, 19), mice with hemizygous deletion of KLF2 (KLF2^±^) are viable. KLF2^±^ mice crossed with ApoE^−/−^ mice are more susceptible to atherosclerotic disease compared with KLF2^+/+^ApoE^−/−^ mice (20). Additionally, post-natal deletion of KLF2 leads to a thrombotic phenotype, while globally overexpressing KLF2 protects mice from thrombus formation in part through the decreased expression of endothelial thrombotic genes (21).

Vascular inflammation is also a major player in the pathogenesis of diabetic vascular disease. Interestingly, hyperglycemia decreases endothelial KLF2 expression *via* FOXO1-dependent transcriptional silencing (22). Moreover, insulin induces KLF2 expression in ECs and KLF2 expression is reduced in the glomerulus of diabetic rats (23). Endothelial KLF2 is also implicated in vascular inflammation seen in neurodegenerative diseases such as Alzheimer’s disease. Amyloid beta plaques, a hallmark of Alzheimer’s, decrease KLF2 levels in cerebral ECs; and overexpression of KLF2 protects against amyloid-induced oxidative stress (24). These studies further demonstrate the protective nature of KLF2 during states of vascular inflammation, expanding the diversity of disease states that would potentially benefit from pharmacological targeting of KLF2.

Vessel hemodynamics strongly influence vascular inflammation and KLF2 is exquisitely sensitive to the biomechanical forces exerted by laminar versus turbulent shear stress. Under conditions of laminar shear stress (LSS), KLF2 is robustly expressed in ECs *in vitro* and *in vivo* (7, 11). Indeed, as recently confirmed by Dekker et al. in humans, KLF2 expression is focally lowered in areas of low LSS such as the bifurcation of the aorta to the iliac and carotid arteries, and this may be downstream of a dual specificity mitogen-activated protein kinase kinase 5(MEK5)/extracellular-signal-regulated kinase 5 (ERK5)/myocyte enhancer factor 2 (MEF2) pathway (7, 25, 26). It has long been known that atherosclerotic lesions have a predilection to form at regions experiencing low LSS, such as bifurcations of the vasculature. Within these so-called “atheroprone” regions, ECs are more likely to become activated and increase production of pro-inflammatory mediators (27, 28).

The extent of KLF2 expression and activity is highly regulated in ECs. As previously mentioned, LSS induces KLF2 expression. The signaling cascade behind this induction has been extensively characterized: In response to LSS, MEK5 is activated, which then phosphorylates ERK5. ERK5 subsequently phosphorylates MEF2 at the KLF2 promoter, leading to KLF2 gene transcription (7, 29). Conversely, KLF2 transcription can be inhibited by tumor necrosis factor alpha (TNF-α) signaling *via* p65 and histone deacetylase (HDAC) 4/5 inhibition of MEF2 (26). p53 also utilizes HDAC5-mediated KLF2 suppression to induce endothelial dysfunction (30). Interestingly, HDAC5 also regulates KLF2’s ability to induce transcription of eNOS, implicating HDACs as regulators of KLF2 function at multiple points (31, 32). Post-transcriptionally, endothelial KLF2 is targeted by microRNA-92a (miR-92a). Low-shear stress and oxidized LDL, factors both associated with atherogenesis, induce miR-92a expression (33, 34). miR-92a is then capable of binding to the 3′-UTR of KLF2, leading to its degradation (33, 35). In fact, targeting miR-92a *in vivo* using an antagomir leads to protection from atherosclerosis, providing a method to indirectly target KLF2 (33). Additional post-transcriptional regulation of KLF2 occurs through PI3K-dependent mRNA stabilization in response to LSS (36).

Krüppel-like factor 2 serves as a prototypical vasoprotective factor as it (1) is induced by EC activating stimuli, (2) resists harmful pro-inflammatory and pro-thrombotic gene transcription, and (3) is associated with protection against vascular inflammatory disease.

### Krüppel-Like Factor 4

Krüppel-like factor 4 shares many transcriptional targets and protective functions with KLF2 in ECs. Like KLF2, KLF4 is induced during LSS (37, 38). Moreover, KLF4 expression is repressed under turbulent flow as a result of DNA methyltransferase-mediated methylation within the KLF4 promoter (39). The same mechanism also silences endothelial KLF3 under turbulent flow, an anti-inflammatory KLF that is less well characterized in ECs (40). Downstream transcriptional effects of KLF4 are similar to those seen in KLF2. For instance, a KLF4–p65 interaction inhibits VCAM-1 induction, reducing leukocyte homing (38, 41). KLF4 also regulates expression of eNOS. In multiple studies, overexpression or knockdown of EC KLF4 leads to increased or decreased eNOS production, respectively (38, 42, 43). KLF4 overexpression also increases transcription of anti-thrombotic factor thrombomodulin as well *via* a physical association with its cofactor, p. 300 (38). A novel and fascinating role of endothelial KLF4 was recently discovered in the context of endothelial inflammation and cholesterol flux. KLF4 induces the expression of cholesterol-25-hydroxylase (Ch25h) and liver X receptor (LXR) in ECs, which contribute to reverse cholesterol transport out of the vascular wall and inhibition of endothelial inflammasome activation, both protective against atherosclerosis (44).

In addition to its role in maintaining an anti-adhesive and anti-thrombotic endothelium, KLF4 also modulates intrinsic EC health. Autophagy is a conserved process by which cells recycle damaged organelles and misfolded proteins. Disrupted autophagy has been associated with multiple age-related phenotypes such as metabolic dysfunction, neurodegeneration, and cardiovascular disease [reviewed in Ref. (45)]. A recent study identified a role for endothelial KLF4 in regulating autophagic genes (46). Endothelial overexpression of *Klf4* protected vessels from vascular aging, an effect that is likely largely due to enhanced autophagy (46). Interestingly, this study also demonstrated an inverse correlation between the age of vessels and KLF4 expression in humans.

The essential role of endothelial KLF4 in vascular health has been demonstrated in multiple *in vivo* models. Endothelial-specific knockout of *Klf4* using a VE-Cadherin driven *Cre*-Lox system results in significantly enhanced atherosclerotic lesions when backcrossed onto the *Apoe*^−/−^ mouse line (38). Additionally, EC-Klf4 KO exhibited increased thrombotic capacity. Conversely, endothelial-driven overexpression of *Klf4* is protective against atherosclerosis. Conversely, endothelial-driven overexpression of Klf4 is protective against atherosclerosis and thrombosis (38). Outside of atherothrombotic disease, vascular inflammation can also negatively affect renal arteries during instances of ischemia. Endothelial KLF4 is vasoprotective in this context as demonstrated in a model of hematopoietic deletion of *Klf4* during ischemia–reperfusion injury (47). Mice lacking KLF4 demonstrated exacerbated renal injury as a result of increased adhesion molecule expression on ECs with consequent immune cell invasion (47). This mechanism was also at play in a model of carotid artery injury. Interestingly, loss of endothelial KLF4 resulted in enhanced proliferation of both EC and neointimal VSMCs, as mediated by increased immune cell presence (41). In another disease model of pathological vascular remodeling, KLF4 levels are decreased in the lungs of patients with pulmonary artery hypertension (PAH) (42). Loss of endothelial KLF4 is associated with increased hypertension and pulmonary artery vascularization, in part through enhanced expression of endothelin-1 (ET-1) and decreased eNOS expression (42).

Like KLF2, KLF4 is post-transcriptionally regulated by miRs. Specifically, both KLF2 and KLF4 are inhibited by the “atheromiR,” miR-92a (33, 35). Additionally, however, KLF4 is negatively regulated by miR-103 (48). In mice with endothelial-specific deletion of miR processing machinery, Dicer, there is a decrease in miR-103-mediated KLF4 suppression; this increase in KLF4 subsequently restrains NF-κB-driven CXCL1 and macrophage infiltration in atherosclerotic lesions (48).

Endothelial KLF2 and KLF4 have remarkably similar functions; they respond to many of the same stimuli, share gene targets and have a high degree of similarity in amino acid sequence. Early studies on endothelial KLF4 function noted overlapping functions between the two phylogenetically close factors (37, 49). Indeed, loss of one allele of *Klf2* leads to a compensatory increase in *Klf4*, while a single allele of either *Klf2* or *Klf4* is sufficient to rescue lethality in a double *Klf2*/*Klf4* knockout mouse, suggesting genetic redundancy of functions central to endothelial function and identity (20, 50). Indeed, the double *Klf2/Klf4* knockout mouse demonstrates loss of endothelial integrity and hemostatic dysfunction, as well as the loss of an endothelial-like transcriptome.

### Krüppel-Like Factors 5 and 6

Unlike KLF2 and 4, endothelial KLF5 and 6 are associated with vascular inflammation and remodeling that is largely deleterious. While KLF5 is largely considered to be a major effector of VSMC function (see below), there is evidence that it mediates endothelial chemotactic function. Specifically, knockdown of endothelial KLF5 *in vitro* reduces TNF-α-induced expression of key monocyte chemoattractant protein, MCP-1 (51). While the *in vivo* implications of this phenomenon are unclear, there is ample evidence implicating MCP-1 in many forms of vascular inflammation including atherogenesis, diabetic vascular disease, and vascular occlusion (52–54).

Largely implicated in cancer biology, KLF6 also has documented roles in ECs. KLF6 is an early response factor to vascular injury that induces transcription and processing of the pro-angiogenic factor endoglin, a member of the TGF-β receptor superfamily member (55). Mechanistically, KLF6 interacts with related transcription factor specificity protein 1 (Sp1) to bind to the endoglin promoter (56). While endoglin’s role in angiogenesis has been extensively characterized, it is also implicated in leukocyte trafficking during vascular inflammation (57). Endothelial KLF6, therefore, may promote immune cell infiltration during vascular injury. In addition to regulating endoglin, KLF6 also induces expression of activin receptor-like kinase 1 (ALK1), another member of the TGF-β receptor family (58). KLF6–Sp1 interactions mediate *Alk1* transcription during endothelial denudation, and KLF6 heterozygotes exhibit reduced neointimal formation in response to vascular injury, a mechanism that is proposed to be through reduced ALK1 levels (58). While additional studies regarding endothelial KLF5 and 6 need to be completed, both factors seem to promote vascular inflammation and remodeling in response to injury.

### Krüppel-Like Factor 11

Krüppel-like factor 11 is also highly expressed in ECs and is involved in regulating vascular inflammation. While *Klf2* expression is ultimately inhibited by TNF-α, KLF11 is induced as a result of inflammation, and similar to interactions seen with endothelial KLF4, KLF11 binds to p65 to inhibit transcription of NF-κB target genes such as VCAM-1 and E-selectin resulting in less leukocyte adhesion to ECs during vascular inflammation (59). This allows KLF11 to serve as an inflammatory-responsive factor to reduce excessive endothelial activation. In fact, loss of KLF11 in a model of cerebral ischemia results in enhanced inflammation and worse outcome (60). Endothelial KLF11 is regulated, in part, by the peroxisome proliferator-activated receptor (PPAR) family of nuclear receptor proteins, which includes three mammalian isoforms: α, β, and γ. Administration of a PPARγ agoinst leads to increased KLF11 expression in cerebral vascular ECs (61). Interestingly, KLF11 also serves as a coregulator of PPARγ target genes *via* physical interaction of the two proteins at PPAR responsive elements (61). This interaction results in repression of pro-apoptotic miR-15a, increasing EC survival and conferring protection against cerebrovascular ischemia. These two studies demonstrate a role of endothelial KLF11 in regulating vascular inflammation during ischemic events. In addition to being responsive to PPARγ, *Klf11* transcription is also induced by PPARα (62). Administration of PPARα agonist causes KLF11 induction and its subsequent binding and inhibition of the ET-1 promoter. This phenomenon further bolsters KLF11’s role as a vasoprotective factor through its ability to inhibit endothelial inflammation and vasoconstriction.

## VSMC KLFs

**Figure 2:**
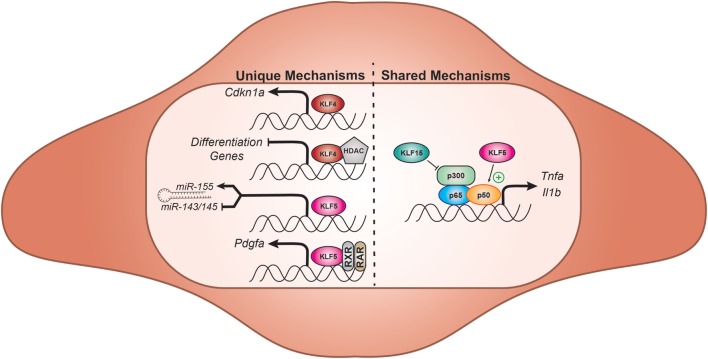
Select effector functions of vascular smooth muscle KLFs. KLFs differentially affect VSMC differentiation, proliferation, and inflammation through a multitude of mechanisms. KLF5 and 15 exhibit reciprocal regulation of nuclear factor-κB signaling within VSMCs by binding to cofactors p50 and p300, respectively. KLF5 also promotes proliferation and dedifferentiation by modulating important driver of VSMC dedifferentiation, platelet-derived growth factor-A (PDGF-A), and differentially regulating miR transcription. While KLF4 also promotes dedifferentiation, it resists VSMC proliferation through the transcription of CDKN1A. KLF, Krüppel-like factor; VSMC, vascular smooth muscle cell; HDAC, histone deacetylase; miR, microRNA; TNF-α, tumor necrosis factor alpha; IL-1β, interleukin-1 beta; RXR, retinoid X receptor; RAR, retinoid acid receptor; Cdkn1a, cyclin-dependent kinase inhibitor 1a.

Vascular smooth muscle cells, along with collagen and elastin, form the medial layer of blood vessels and regulate vasomotor tone to maintain proper hemodynamic pressure throughout the vascular system. In the absence of noxious stimuli, VSMCs express numerous mature markers including smooth muscle α-actin, SM22α, and smoothelin (63). When challenged by growth factors, inflammation, or injury, VSMCs undergo phenotypic switching: cells dedifferentiate, losing mature VSMC markers and regaining the ability to proliferate, migrate, and synthesize extracellular matrix proteins. This can lead to pathological vessel remodeling, ultimately resulting in obstruction of proper blood flow. Multiple KLFs modulate VSMC phenotype switching in the face of vascular injury and inflammation (Figure 2).

### Krüppel-Like Factor 4

Vascular smooth muscle cell KLF4 maintains cells in a dormant state by binding to and recruiting the potent anti-proliferative protein p53 to the p21^WAF1/Cip1^ (*Cdkn1a*) promoter/enhancer, consequently increasing transcription of *Cdkn1a*, a cell cycle inhibitor (64, 65). Further, viral overexpression of KLF4 in both VSMC and balloon-injured rat carotid arteries leads to increased expression of a panel of anti-proliferative genes such as p57 and GADD45β (66). *In vivo* implications of KLF4’s anti-proliferative effects are seen in a carotid artery ligation model using conditional KLF4 knockout: KLF4 KO mice demonstrate enhanced neointimal formation as a result of dysregulated VSMC proliferation (64).

Given VSMC KLF4’s role in suppressing proliferation, KLF4 also plays a somewhat counterintuitive role in promoting dedifferentiation of VSMCs. KLF4 coordinates multiple molecular events to repress markers of VSMC maturity in the context of pathological vessel remodeling. KLF4 binds directly to the TGF-β control element (TCE) to inhibit transcription of smooth muscle α-actin and SM22α (64, 67). Moreover, KLF4 recruits inhibitors of mature VSMC marker transcription such as ELK-1 and HDACs to TCEs (68). Recent evidence indicates that VSMC dedifferentiation during vascular injury not only decreases markers of mature VSMCs, but also causes VSMCs to express markers associated with macrophages, myofibroblasts, and mesenchymal stem cells. Utilizing elegant lineage tracing experiments, Shankman et al. showed that KLF4 is necessary for VSMCs to gain genetic characteristics of other cell types within atherosclerotic lesions (69). Evidence for this transition from VSMC to a “macrophage-like” cell type was recapitulated *in vitro* using cholesterol loaded *Klf4-*sufficient VSMCs, an effect that was lost in *Klf4* mutant cells (69). These studies demonstrate KLF4’s importance in VSMC phenotype switching during pathological remodeling. Modulating KLF4 expression may provide an important therapeutic avenue to adjust VSMC phenotype and disease progression.

Mature, uninjured VSMCs express very low levels of KLF4 both *in vitro* and *in vivo* (67, 70); however, KLF4 expression is significantly increased in injured VSMCs, contributing to subsequent dedifferentiation. Oxidized phospholipids, which are associated with atherosclerotic burden, induce both KLF4 mRNA transcription and KLF4 nuclear translocation in VSMCs (71). Additionally, carotid artery ligation-induced vascular injury leads to a swift increase in VSMC KLF4 expression that is associated with repression of smooth muscle α-actin (64). Mechanistically, this occurs through the reduction of miR-143/145, which normally inhibit KLF4 expression to promote VSMC maturation (72). Cigarette smoke is another well-characterized stimulant of vascular inflammation. Cigarette smoke extract has been shown to induce *Klf4* transcription, enhance KLF4 binding at the promoters of VSMC differentiation genes, and increase KLF4-driven epigenetic changes that are associated with transcriptional repression (73).

### Krüppel-Like Factor 5

Krüppel-like factor 5 modulates processes that engender pathological remodeling of the vasculature: like KLF4, KLF5 promotes VSMC dedifferentiation; however, dissimilarly, KLF5 also promotes cellular proliferation. KLF5’s importance in phenotype switching in the face of vascular injury has been shown both *in vitro* and *in vivo*. Overexpression of KLF5 leads to reduced expression of VSMC maturity markers myocardin and smooth muscle α-actin while the converse occurs with KLF5 knockdown (74). Concordantly, *in vivo* experiments show that in wild-type mice, vascular injury causes a decrease in smooth muscle α-actin and smooth muscle myosin heavy chain (MHC), whereas this effect is not seen in mice heterozygous for KLF5 (75).

Krüppel-like factor 5’s exerts its pro-inflammatory effects through two main mechanisms: complex formation with unliganded retinoid acid receptor (RAR)/retinoid X receptor (RXR) and recruitment of NF-κB subunit p50. KLF5-RAR/RXR complex binds to the promoter of platelet-derived growth factor-A (PDGF-A), a potent inducer of VSMC proliferation and dedifferentiation (75, 76). Administration of a synthetic retinoid abolishes KLF5-RAR/RXR interaction, resulting in decreased KLF5 transcriptional activity; whereas, administration of an RAR antagonist stabilizes KLF5 transcriptional effects (75, 77). Furthermore, this mechanism has been shown *in vivo* to govern VSMC proliferation in times of injury. Both pharmacological KLF5 inhibition *via* the administration of an RAR agonist and genetic loss of one *Klf5* allele diminishes neointimal formation and vascular remodeling in models of AngII infusion or femoral artery cuff injury, indicating a critical role of KLF5 in VSMC proliferation in response to injury (77). In addition to KLF5-RAR/RXR complex formation, KLF5 recruits p50 to influence VSMC phenotype toward proliferation and dedifferentiation, as well as augment VSMC inflammatory transcription (78, 79). In VSMCs from diabetic patients, increased *KLF5* and inducible nitric oxide synthase expression leads to augmented nitrated-KLF5, which possesses a heightened ability to interact with p50 and subsequently enhance TNF-α and interleukin-1 beta (IL-1β) expression (79). Interestingly, estradiol competes with KLF5 for p50 binding and can inhibit KLF5-p50-mediated transcription of inflammatory genes (79).

Krüppel-like factor 5 has been found to be upregulated in both human lung biopsies and isolated human pulmonary artery smooth muscle cells from patients with PAH, a vascular remodeling disease process (80). Using a hypoxic PAH model, Li et al. demonstrated that KLF5 serves as an upstream regulator of hypoxia inducible factor 1-alpha activity (81). Loss of KLF5 abrogated hypoxia-induced vascular remodeling partly through upregulating proliferation factors (e.g., cyclin B1 and D1) and downregulating apoptosis factors (e.g., bax, bcl-2, cleaved caspase-3, and cleaved caspase-9) (80, 81). A similar effect is seen in cardiomyocytes in response to ischemia/reperfusion injury, further emphasizing a conserved role of KLF5 in promoting proliferation and cell survival (82).

Similar to other KLFs, VSMC KLF5 both regulates and is regulated by the transcription of miRs. miR-145 is highly expressed in differentiated VSMCs and is important in maintaining cellular differentiation: expression of miR-145 is associated with upregulation of smooth muscle α-actin, calponin, and smooth muscle-MHC and reciprocal downregulation of KLF5 (72, 74, 83). In injured vessels such as those seen in atherosclerotic lesions, however, expression of miR-145 is downregulated within VSMCs (74, 84). In the absence of vascular injury, miR-145 directly targets the 3′-UTR of KLF5 to inhibit it. Following injury, PDGF inhibits miR-145 expression, thus attenuating KLF5 degradation and consequently suppressing transcription of differentiation markers (see above) and myocardin, a modulator of differentiation genes (74). In addition to being regulated by miRs, KLF5 also controls the expression of the pro-inflammatory miR-155. When VSMCs are exposed to oxLDL, KLF5 is induced, resulting in decreased anti-inflammatory miRs-143/145 and increased miR-155 (85). Interestingly, KLF5-mediated miR-155 production leads to secreted miR-155 in exosomes that are capable of destroying endothelial tight junctions and enhancing atherosclerotic progression (85).

### Krüppel-Like Factor 15

Krüppel-like factor 15 serves largely as a protective factor in many aspects of cardiovascular biology including inhibiting cardiomyocyte hypertrophy, regulating cardiac lipid metabolism, and establishing circadian control of ventricular rhythm (86–88).

Vascular smooth muscle cell KLF15 primarily acts as a protective factor against vascular inflammation and disease by resisting VSMC proliferation and inflammation. Similar to other KLFs, VSMC KLF15 interacts with the histone acetylase, p300. Direct binding to p300 prevents acetylation of NF-κB member p65, thus limiting transcription of NF-κB target genes and inflammation. Both rat aortic VSMCs exposed to oxidized phospholipids and human atherosclerotic tissue demonstrate markedly decreased KLF15 expression, suggesting that KLF15 plays an important role in atherogenesis (89). Orthotopic carotid artery transplantation from Klf15^−/−^ mice into *Apoe*^−^*^/^*^−^ mice results in significantly enhanced intimal hyperplasia and inflammatory cell infiltrate. Additionally, these VSMCs express higher levels of inflammatory proteins such as VCAM-1, MCP-1, and MMP3. These results have been recapitulated in smooth muscle-specific deletion of *Klf15* on the *Apoe*^−^*^/^*^−^ background. When rat aortic smooth muscle cells are exposed to PDGF-BB, a stimulator of VSMC proliferation and migration, *Klf15* mRNA expression is reduced (90). Interestingly, in KLF15-deficient VSMCs, *Pdgf* transcription is enhanced, demonstrating a feed-forward loop that permits VSMC proliferation and inflammation by decreasing KLF15 levels (89). In humans and mice, KLF15 deficiency is associated with cardiomyopathy and aortic aneurysm (91). Additionally, humans with ruptured intracranial aneurysms exhibit diminished KLF15 expression while expressing elevated levels of inflammatory genes (92).

## Monocyte/Macrophage KLFs

**Figure 3:**
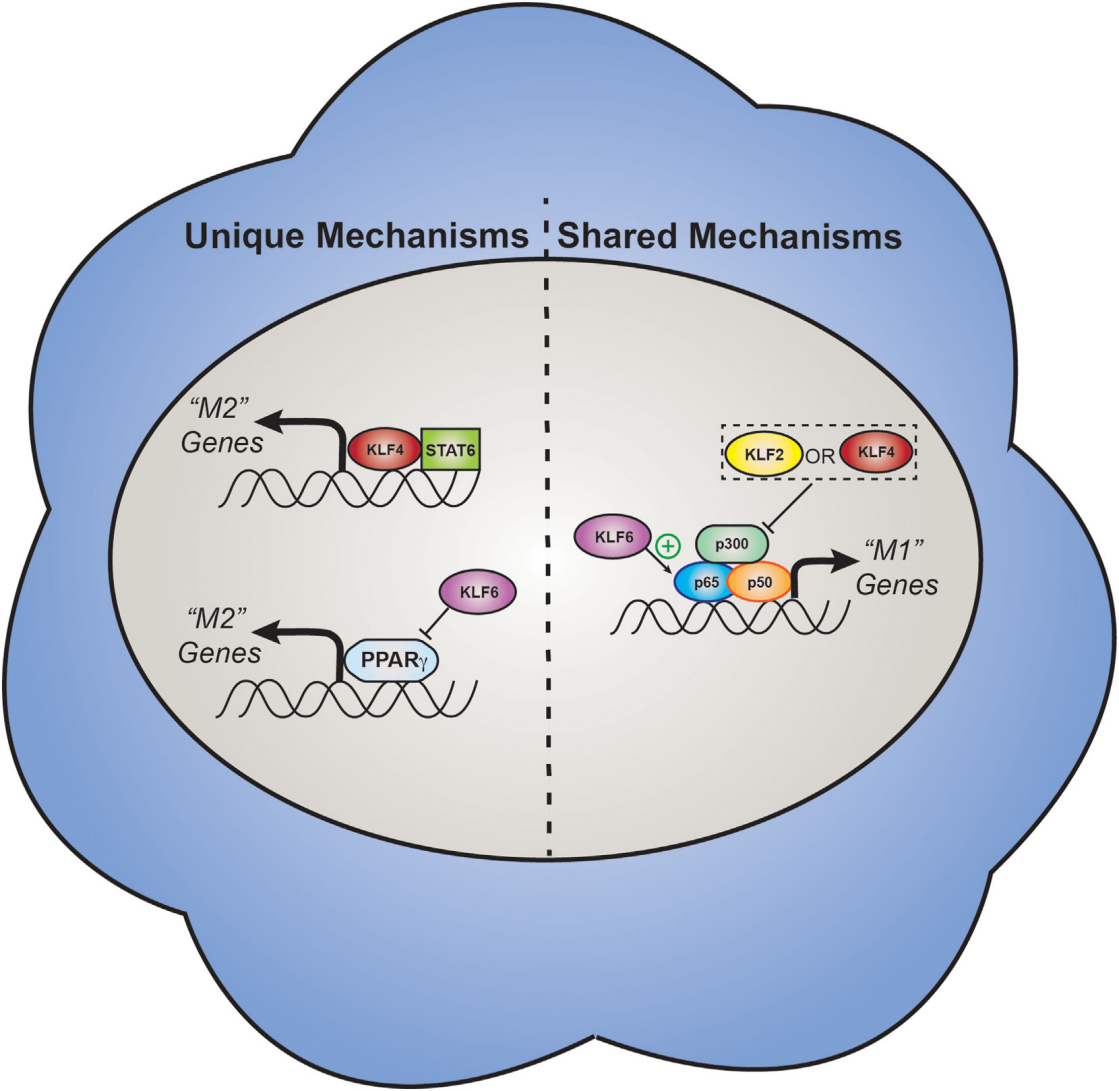
Select effector functions of myeloid KLFs. Myeloid KLFs affect polarization of cells to a pro-inflammatory (“M1”) or anti-inflammatory (“M2”) state. KLF2 and 4 inhibit M1 polarization by negatively regulating nuclear factor (NF)-κB-mediated transcription. Additionally, KLF4 promotes M2 polarization through its interactions with STAT6, a prominent transcription factor in M2 skewing. KLF6 promotes M1 polarization through cooperation with NF-κB cofactor p65 and through its inhibition of PPARγ-mediated M2 transcription. KLF, Krüppel-like factor; miR, microRNA; PPARγ, peroxisome proliferator-activated receptor gamma; M1, pro-inflammatory classical macrophage activation; M2, anti-inflammatory alternative macrophage activation; STAT6, signal transducer and activator of transcription 6.

In addition to cells of the vessels themselves, circulating immune cells and their infiltration into the vascular wall are paramount to the initiation and propagation of vascular inflammation. There is ample research implicating both innate and adaptive immune cells in the progression of atherosclerosis [reviewed in Ref. (93, 94), respectively]. Comparable with their role in vascular cells, KLFs have divergent functions in myeloid cell-derived inflammation, capable of either repressing or promoting inflammatory processes (Figure 3).

### Krüppel-Like Factor 2

Originally studied in the context of acute inflammation and bacterial sepsis, KLF2 is a central regulator of monocyte inflammatory activation (95, 96). KLF2 resists inflammation within macrophages *via* recruitment of NF-κB cofactors away from the promoters of inflammatory genes. Among these factors bound by KLF2 are p300 and p300/CBP-associated factor (PCAF) (95, 97, 98). Given the inflammatory potential of KLF2 knockout macrophages, one would predict that loss of myeloid KLF2 would be associated with increased vascular inflammation and atherosclerosis. This is, indeed, the case as mice with myeloid-specific KLF2 deletion on the *Apoe*^−^*^/^*^−^ background exhibit increased atherosclerosis with increased vascular oxidative stress (99). This is the result of increased adhesive potential of KLF2 knockout neutrophils and macrophages. Interestingly, a similar effect is seen with dendritic cell (DC)-specific KLF2 knockout. Loss of KLF2 in DCs aggravates atherosclerosis as a result of enhanced T-cell activation and heightened inflammatory cytokine production (100). Together, these studies demonstrate a central role of KLF2 in maintaining quiescence in circulating myeloid cells; a role it serves in ECs as well.

Krüppel-like factor 2 is itself controlled by atherogenic stimuli. When anti-inflammatory macrophages are challenged with oxLDL, they shift to a pro-inflammatory state *via* the downregulation of KLF2 (101). There is also a link between low-immune cell KLF2 levels with increased risk of cardiovascular disease in humans. Monocytes from patients with atherosclerosis exhibit less *Klf2* expression than healthy controls, indicating that the inflammatory state associated with low-KLF2 translates to atherosclerotic disease (98).

### Krüppel-Like Factor 4

Just as KLF2 and KLF4 have overlapping functions in ECs, this is also the case in monocytes/macrophages. While KLF2 regulates inflammatory activation of monocytes, KLF4 regulates macrophage polarization from the pro-inflammatory (“M1”) state to the anti-inflammatory (“M2”) state (102). Like KLF2, KLF4 recruits p300 and PCAF away from the promoter of inflammatory genes, resisting the M1 polarization state. Complementarily, KLF4 promotes the M2 state by cooperating with critical M2 transcription factor STAT6 to induce transcription of traditional M2 genes through induction of MCP-1-induced protein (102, 103). While STAT6 is largely responsible for anti-inflammatory polarization in macrophages, CREB is another transcription factor that limits resists inflammation (104). In addition to interacting with STAT6 to modulate anti-inflammatory transcription, KLF4 also interacts with CREB to increase transcription at the apoE promoter in macrophages, ultimately resulting in atheroprotection (105–107). Myeloid KLF4, therefore, resists inflammation and is largely a protective factor against vascular inflammation. Moreover, loss of myeloid KLF4 is associated with augmented atherosclerosis, and macrophages deficient in KLF4 display increase inflammation in response to oxidized phospholipids (108). While KLF4-mediated Ch25h and LXR expression drives reverse cholesterol transport in ECs and macrophages, there is also evidence implicating Ch25h and LXR in KLF4-mediated M2 polarization (44). It is evident that KLF4 regulation of macrophage polarization and its role in preventing vascular inflammation is exceedingly complex and likely involves multiple downstream regulators.

### Krüppel-Like Factors 5 and 6

The increased inflammatory drive associated with endothelial KLF5 and 6 is paralleled by that of macrophage KLF5 and 6. Overexpression of KLF5 increases the ability of macrophages to migrate and proliferate (109). This contributes to worsened intimal hyperplasia following carotid ligation in KLF5 overexpressing mice. This is in contrast to the protective effect afforded by myeloid-specific KLF5 deletion. Interestingly, pro-inflammatory stimuli stabilize KLF5 protein *via* various post-translational modifications. TNF-α increases KLF5 sumoylation and decreases ubiquitination to stabilize the protein and prevent degradation (109, 110). KLF5’s responsiveness to inflammatory stimuli, along with its ability to propagate macrophage-mediated inflammation, contributes to its deleterious role in vascular inflammation.

Krüppel-like factor 6 expression is also responsive to pro-inflammatory stimuli. KLF6 increases when macrophages are stimulated with M1-driving stimuli and decreases with M2-driving stimuli (111). Additionally, KLF6 impacts both M1 and M2 gene transcription. KLF6 is required for optimal binding of p65 binding to its promoters, and importantly, through its interaction with p65, KLF6 promotes transcription of NF-κB targets (112). Additionally, KLF6 suppresses B cell lymphoma 6 expression, which leads to increased pro-inflammatory gene expression and increased macrophage motility (113). Conversely, KLF6 binds to PPARγ and prevents it from inducing M2 gene transcription (111). It is evident that KLF6 is a dynamic regulator of macrophage polarization.

### Krüppel-Like Factor 14

Within the past 5 years, KLF14 has been extensively studied in its role in lipid and cholesterol metabolism. Given that aberrant nutrient handling, obesity, and type 2 diabetes are risk factors for atherosclerosis, it is unsurprising that multiple genetic variants involving the *KLF14* gene have been implicated in the development of atherosclerotic disease. While the genetic associations of *KLF14* variants on metabolic disease have been extensively studied (114), the role of KLF14 in macrophages is less well characterized. Recent work by Wei and colleagues has begun to parse out details on how KLF14 contributes to atherogenesis. They found that *Apoe*^−^*^/^*^−^ mice aortas had elevated levels of *Klf14* on either high-fat diet or standard chow (115). This increase in Klf14 expression was associated with elevated pro-inflammatory cytokines in circulation: *Klf14* adenoviral knockdown ameliorated this effect. Importantly, overexpression of *Klf14* in a macrophage cell line led to increased inflammatory cytokine production as well as total cholesterol and cholesteryl ester content, a classic signature of atherogenic foam cells (116). Mechanistically, KLF14-mediated inflammation seems to be dependent on p38 MAPK and ERK1/2 signaling leading to increased cytokine release (115). Together, this work provides evidence that KLF14 may play a causal role in modulating inflammation associated with atherosclerosis, further implicating it in metabolic disease.

## KLFs in Other Circulating Immune Cells

**Figure 4:**
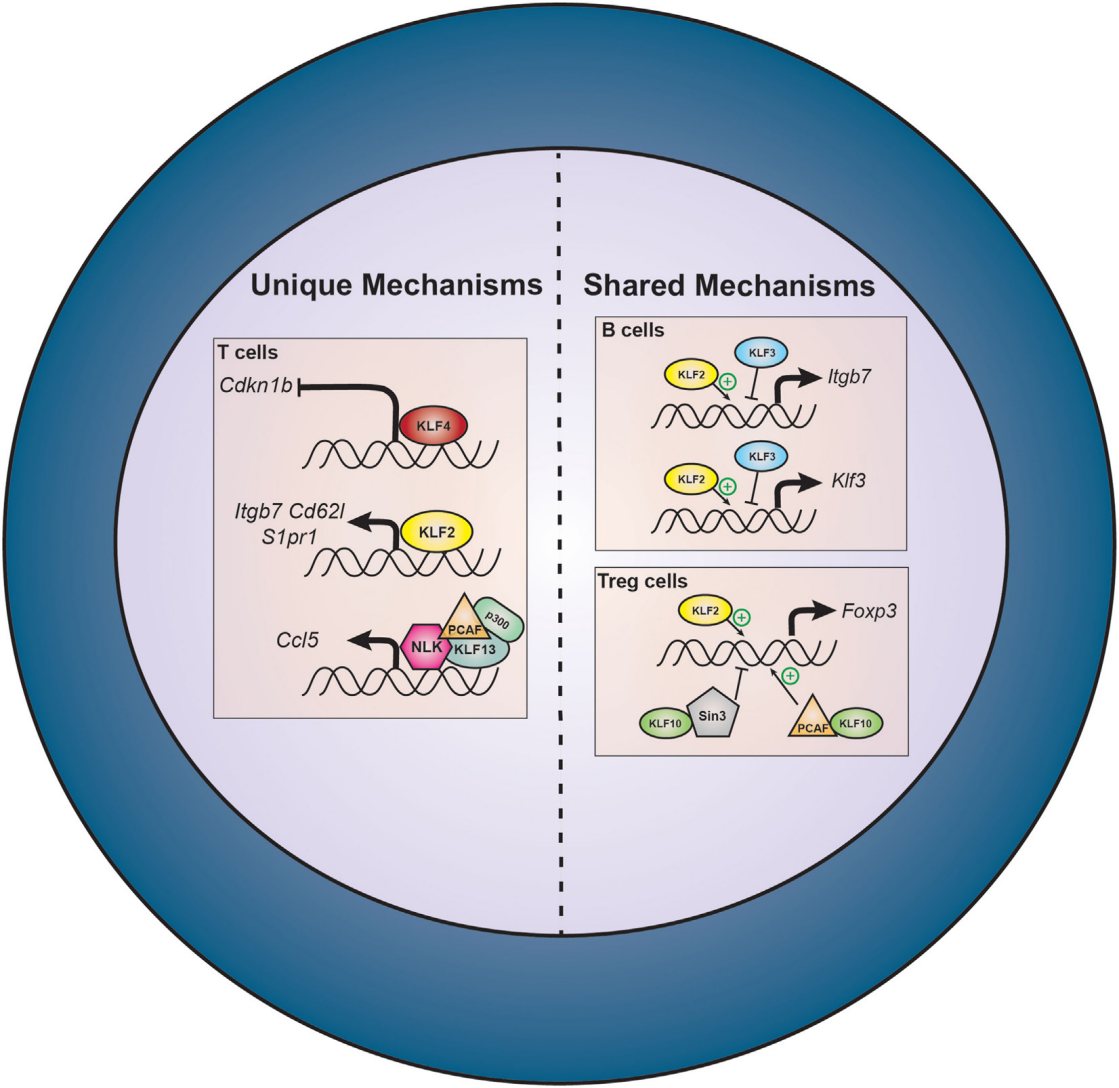
Select effector functions of lymphocyte KLFs. Lymphocyte KLFs largely dictate cellular identity, proliferation, and survival. KLF2 and KLF3 oppose each other’s function at the *Itgb7* and *Klf3* promoters. In regulatory T cells (Treg), corepressor/coactivator interactions determine the transcriptional effects of KLF10. KLF, Krüppel-like factor; Itgb7, β_7_ integrin; Foxp3, forkhead box P3; Sin3, represents Sin3-histone deacetylase complex (Sin3-HDAC); PCAF, P300/CBP-associated factor; Cdkn1b, cyclin-dependent kinase inhibitor 1b (p27^Kip1^); Cd62l, cluster of differentiation 62 ligand (L-selectin); S1pr1, sphingosine-1-phosphate receptor 1 (S1P_1_); Ccl5, chemokine ligand 5 (RANTES); NLK, Nemo-like kinase.

In addition to regulating differentiation, activation, and polarization of monocytes, KLFs also shape lymphocyte and DC function. While there is a paucity of studies investigating KLF-driven lymphocyte processes in vascular inflammation, there is extensive evidence demonstrating the importance of KLFs in lymphocyte biology that can be extrapolated to the context of vascular disease (Figure 4).

### Krüppel-Like Factor 2

Like in monocytes, KLF2 expression maintains T cells in a quiescent state. KLF2 is expressed in naïve, effector, and memory T cells (117, 118) and its loss causes single-positive, resting T cells to spontaneously activate and apoptose in the spleen and lymph nodes (18, 19). In CD8+ T lymphocytes, KLF2 levels decrease upon stimulation of T-cell receptors and its expression is reestablished after treatment with IL-2 or IL-7 (118). In contrast, CD4+ T lymphocytes demonstrate a transient increase in KLF2 expression upon stimulation that is associated with increased IL-2 production (119). KLF2 is essential in the expression of T-cell migration factors such as S1P_1_, cluster of differentiation 62 ligand (L-selectin) (CD62L), and β_7_ integrin (Itgb7), allowing T cells to traffic to sites of vascular inflammation or draining lymph nodes (120, 121). Furthermore, statin-induced KLF2 expression in effector T cells reduces inflammation in a myocarditis model, an effect that is likely related to diminished interferon-γ production (122).

In addition to the proatherogenic functions of effector T cells, regulatory T cells (Tregs) play an important role in suppressing vascular inflammation (123). In the presence of oxLDL, Tregs restore *endothelial* KLF2 to protect the vasculature from inflammation (124). Within Tregs, forkhead box P3 (FoxP3), a lineage-specific transcription factor, is under direct control of KLF2 (125). Loss of KLF2 prior to FoxP3 induction results in impaired Tregs differentiation, while loss of KLF2 after FoxP3 induction does not affect this process. Pabbisetty et al. also demonstrated that stabilization of KLF2 protein through statin administration or by genetic deficiency of E3 ubiquitin ligase SMURF1 results in enhanced Treg production.

Within B lymphocytes, KLF2 appears to be important in determining cellular identity. Higher KLF2 expression is associated with B1 B cells in the periphery versus follicular or marginal zone B cells. Concordantly, inactivation of KLF2 in B cells leads to a decrease in B1 B cells with a concurrent increase in marginal zone B cells (126, 127). Similar to T cells, loss of KLF2 in B cells is also associated with less CD62L and Itgb7 expression, resulting in impaired B-cell trafficking (126, 128). Finally, KLF2 also plays a role in regulating the DC response during vascular inflammation. As is seen with monocytes and neutrophils (99), loss of KLF2 in DCs increases inflammatory cytokine production, DC tissue infiltration, and T-cell activation in atherogenic *Ldlr*^−^*^/^*^−^ mice (100). Together, these studies further demonstrate that KLF2 largely opposes inflammatory activation in circulating immune cells.

### Krüppel-Like Factor 3

Within B cells, KLF2 and KLF3 have opposing effects and compete for the same gene targets. While KLF2 is associated with a B1 B-cell differentiation pattern (with lower levels associated with follicular and marginal zone cells), KLF3 expression favors marginal zone B-cell development (129). Additionally, KLF2 and KLF3 compete for occupancy of the *Itgb7* promoter: while KLF2 promotes expression of Itgb7 and, thus, migration, KLF3 leads to downregulation of Itgb7 and impaired homing ability of lymphocytes (130). Interestingly, KLF2 and 3 differentially regulate KLF3 expression itself. KLF3 negatively regulates its own expression through direct binding to the KLF3 promoter (130). Conversely, loss of KLF2 in B cells results in decreased expression of KLF3 (i.e., KLF2 increases KLF3 expression) (128). The interplay between these two factors is critical for B-cell differentiation and function.

### Krüppel-Like Factor 4

Given its well-defined role in maintaining self-renewing capabilities of stem cells, it is unsurprising that loss of KLF4 expression is necessary for proper T-cell development. Remarkably, KLF4 is the only Yamanaka factor that is downregulated throughout each step of T-cell differentiation (131). This attenuation is required for the transition from double negative (DN)2 to DN3 as evidenced by diminished T-cell differentiation at this stage during forced KLF4 overexpression (131). While loss of KLF4 is critical to T-cell *differentiation*, DN T-cell population proliferation is maintained through KLF4 activity: KLF4 binds to and inhibits the promoter of cyclin-dependent kinase inhibitor 1b/p27^Kip1^, releasing inhibition of CDK-mediated proliferation (132). Interestingly, this is contrary to how KLF4 interacts with p27^Kip1^ in VSMCs [(66); see below], demonstrating cell-type specific functions of KLF4 in regulating proliferation. Within B cells, KLF4 is lowly expressed in the most immature stages but is increased throughout B-cell maturation (133). Upon activation, however, mature B cells decrease KLF4 levels. Additionally, KLF4 appears to be important in promoting B-cell proliferation through the activation of cyclin D2 (133). KLF4’s role in DC biology closely mirrors that seen in monocyte/macrophages. IRF4-expressing DCs are important in promoting type 2 helper T-cell (Th2) response, and KLF4 is required for this interaction (134). Additionally, loss of KLF4 in pre-DCs leads to fewer IRF4-expressing DCs. Together, the DC and monocyte data suggests that KLF4 strongly favors the “anti-inflammatory” polarization of immune cells and its expression may be a potential target to reduce deleterious vascular inflammation.

### Krüppel-Like Factor 10

Krüppel-like factor 10 plays an important role in establishing Treg identity through FoxP3 expression while also directly promoting Treg function through TGFβ1 production. Indeed, forced overexpression of KLF10 in CD4+ CD25− (non-Treg) T cells induces both *Foxp3* and *Tgfb1* expression while downregulating markers of Th1 and Th2 cells (Tbet and Gata3, respectively) (135). Conversely, loss of KLF10 in CD4+ CD25− cells enhances Th1 and Th2 differentiation. In response to Treg stimulating factor TGFβ1, KLF10 transactivates both FoxP3 and TGFβ1 promoters, representing a positive feedback loop of Treg function (135). Important in vascular inflammation, the addition of KLF10 knockout CD4+ CD25− T cells promoted atherosclerosis in ApoE^−/−^/scid/scid mice *via* increased leukocyte accumulation and inflammatory cytokine production (135). Recent studies have provided mechanistic insight on how KLF10 regulates FoxP3 transcription. Within Tregs, KLF10 recruits PCAF to the FoxP3 promoter, leading to acetylation and subsequent activation of the FoxP3 promoter (136). Remarkably, KLF10 also associates with the corepressor Sin3-HDAC to repress FoxP3 transcription. A study by Xiong et al. demonstrated that PCAF disrupts KLF10/Sin3 interactions to allow PCAF-mediated FoxP3 acetylation through its interaction with KLF10 (137). The authors of this study posit that KLF10 interacts with Sin3-HDAC in the dominant state while post-translational modifications in KLF10 downstream of lymphocyte signaling is required to favor PCAF/KLF10 interactions. Along these lines, KLF10 interaction with the FoxP3 promoter appear to be dependent on Itch-mediated ubiquitination of KLF10 in a degradation-independent manner (138). While this study did not investigate how ubiquitination of KLF10 affects PCAF or Sin3 interactions, ubiquitination of KLF10 promoted FoxP3 expression suggesting that this mechanism may contribute to KLF10’s interaction with PCAF.

### Krüppel-Like Factor 13

Krüppel-like factor 13 also demonstrates complex interactions with acetyl transferases/deacetylases to regulate activation of T cells. RANTES (or chemokine ligand 5) is a classically expressed gene late in T-cell activation whose blockade is associated with diminished atherosclerosis (139). KLF13 promotes RANTES expression through the recruitment of a “enhancesome” that consists of various kinases and acetyltransferases. Specifically, Nemo-like kinase is recruited to phosphorylate the H3 histone on the RANTES promoter. Following this, PCAF and CBP/p300 are recruited to acetylate H3 and allow for ATP-dependent chromatin remodeling and RNA Polymerase II binding (140, 141). In addition to promoting RANTES expression in T cells, KLF13 also promotes apoptosis by binding to the promoter of anti-apoptotic factor BCL-X_L_ and reducing its expression (142). The authors of this study suggest that KLF13-mediated repression of BCL-X_L_ occurs through the recruitment of Sin3-HDAC to the promoter, as is seen in the context of other genes inhibited by KLF13 (143).

## Pharmacological Modulation of KLFs

Current therapies for atherosclerosis largely target mechanisms known to activate vascular inflammatory cascades such as dyslipidemia (statins), disturbed flow (anti-hypertensives), and activated circulating inflammatory cells (aspirin). Given the importance of these stimuli in the pathogenesis of atherosclerosis and thrombosis, understanding molecular mediators of vascular inflammation is imperative in developing novel agents against cardiovascular disease.

**Figure 5:**
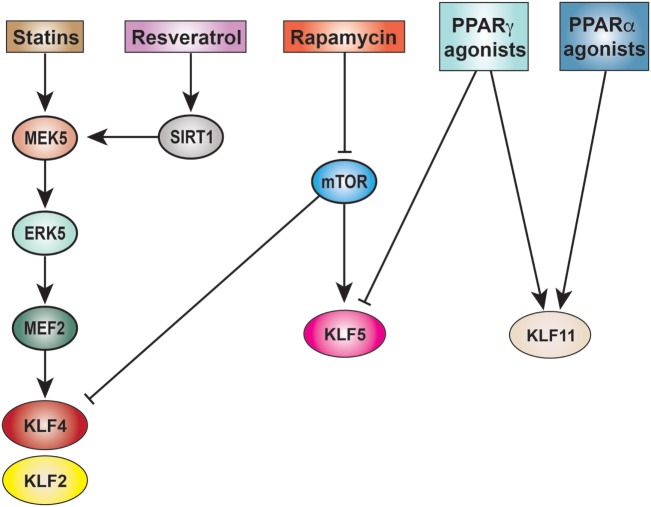
Pharmacological targeting of KLFs in vascular inflammation. There is substantial crossover in the mechanisms of drugs targeting the induction/inhibition of KLFs in vascular inflammation. Both statins and resveratrol augment KLF2 through MEF2-mediated transcription, the same pathway used in statin-mediated KLF4 induction. Rapamycin has differential effects on KLF4 and KLF5 expression, eventually stimulating KLF4 production while inhibiting KLF5. Inhibition of KLF5 is also achieved through use of PPARγ agonists (e.g., rosiglitazone). KLF11 is induced through PPAR signaling, exhibiting increased expression in response to agonists to both PPARγ and PPARα (e.g., fenofibrate). KLF, Krüppel-like factor; MEK5, dual specificity mitogen-activated protein kinase5; ERK5, extracellular-signal-regulated kinase 5; MEF2, myocyte enhancer factor 2; SIRT1, sirtuin 1; mTOR, mammalian target of rapamycin; PPAR, peroxisome proliferator-activated receptor.

While accomplishing specificity in targeting KLFs will likely be difficult, multiple compounds act upstream of KLFs, thereby modulating their expression and function (Figure 5). Below, we summarize a few modulators of KLF biology, with special emphasis on those that affect multiple different KLFs important in vascular inflammation.

### Krüppel-Like Factor 2

Numerous pharmacological agents induce KLF2. Notably, the prominent lipid-lowering statins are potent inducers of KLF2 expression in ECs and circulating immune cells *via* MEF2 (144–146). Studies in mouse have demonstrated a potential role for statin-induced KLF2 expression in protecting against diabetic vascular reactivity and inflammation, as well as myocarditis (122, 147). These studies indicate the widespread anti-inflammatory properties of statins through the modulation of KLF2. In addition to statins, phenol compounds such as tannic acid and resveratrol, are capable of inducing endothelial KLF2 and preventing inflammation (148, 149). Acting *via* sirtuin 1 and MEK5/MEF2-dependent mechanisms, resveratrol induces the expression of KLF2-dependent atheroprotective genes (149). While the benefits of chronic resveratrol therapy in humans are still under investigation, it has been attributed with increasing lifespan and the prevention of multiple age-related diseases in small mammals [(150, 151)]. Additional work needs to be done, however, to determine the relative contribution of KLF modulation in resveratrol’s protective qualities.

Therapeutic proteasome inhibitor Bortezomib has also been demonstrated to induce KLF2 in multiple cell types (21). Normally prescribed to combat multiple myeloma, Bortezomib treatment at non-myelosuppresive doses is actually thromboprotective, in part, through KLF2 induction (21).

### Krüppel-Like Factor 4

As with KLF2, KLF4 expression is induced by statin use. Utilizing a MEK5/ERK5 axis, statin-induced KLF4 expression leads to increased transcription of genes associated with anti-thrombosis, vasodilation, and hemostasis while increasing apoptosis resistance and decreasing inflammatory potential in ECs (43). Additionally, in a model of renal ischemia–reperfusion injury, statins protected against injury in a KLF4-dependent manner (47). Given their widespread use in patients at risk for cardiovascular disease, statins represent a tool to further understand the importance of KLFs in regulating vascular inflammation in humans.

Vascular smooth muscle cell KLF4 is also induced by multiple pharmacological agents including rapamycin and cyclosporine A (CSA). Rapamycin is a known inhibitor of cell proliferation *via* induction of p27^kip1^ (152) and has long been used in drug-eluting stents to prevent restenosis *via* VSMC proliferation (153). Within VSMCs, rapamycin inhibits mammalian target of rapamycin (mTOR), which subsequently increases KLF4 production (66). Interestingly, overexpression of KLF4 results in increased p27^kip1^ production and inhibition of VSMC proliferation. These results suggest that rapamycin and VSMC KLF4 enhance each other’s activities in the regulation of VSMC proliferation. CSA is an immunosuppressant used in inhibiting lymphocyte proliferation that upregulates VSMC KLF4 production, resulting in anti-proliferative and phenotype switching effects (154).

### Krüppel-Like Factor 5

Contrary to its inductive effect on endothelial KLF2, resveratrol has been shown to decrease TGF-β-mediated KLF5 transcription (155). Through its inhibition of the Akt-mTOR pathway, resveratrol is capable of blocking KLF5-driven VSMC dedifferentiation, thereby preventing intimal hyperplasia. Additionally, targeting this TGF-β/phospho-Akt/phospho-mTOR/KLF5 axis with Akt inhibitor LY249004 or mTOR inhibitor rapamycin also decreases KLF5 levels. As previously mentioned, retinoid agonists and antagonists can also diminish and augment KLF5 activity, respectively, by targeting processes downstream of KLF5-mediated transcription. A recent study demonstrated that the PPARγ agonist, rosiglitazone, is capable of reducing VSMC proliferation by suppressing KLF5 expression (156). While PPAR agonists have differential effects on KLF expression (see KLF11 below), their importance in modulating KLF activity cannot be understated as they represent critical modulators of vascular inflammation. Interestingly, there is also evidence that the traditional Chinese medicine Tongxinluo inhibits macrophage KLF5 transcription and blocks PI3K/Akt signaling to prevent KLF5 sumoylation (109).

### Krüppel-Like Factor 10

Given its importance in Treg homeostasis, targeting KLF10 is a potential therapeutic option to combat vascular inflammation. Interestingly, a screen investigating small molecule *inhibitors* of KLF10 identified multiple compounds that are able to prevent conversion of CD4+ CD25− T cells to CD4+ CD25+ Tregs (157). While this was done in the context of reducing Treg effects in immunosuppression seen in cancer, it is feasible that a similar screen can be utilize to identify small molecule *activators* of KLF10 to be used in inflammatory conditions.

### Krüppel-Like Factor 11

Krüppel-like factor 11 is under the transcriptional control of PPAR nuclear receptors and its expression and activity can be indirectly targeted through the use of PPAR agonists. The PPARα ligand fenofibrate stimulates KLF11 transcription and, therefore, inhibits ET-1 production (62). In addition, fenofibrate has demonstrable beneficial effects in preventing diabetic microvascular complications (158). Taken together, KLF11 targeting may serve as a potential mechanism of vascular protection during PPARα agonist use. Pioglitazone, a PPARγ agonist, has cytoprotective properties in cerebrovascular ECs *in vitro* and *in vivo* (61). In the absence of KLF11, however, these effects are lost, indicating a dependency of pioglitazone on KLF11.

## Concluding Remarks

Vascular inflammation is central to the pathogenesis of a wide array of debilitating conditions, especially those most prominent in Western society. Inflammatory responses in the vessel wall and circulating cells are governed, in part, through the action of select transcriptional regulators with a body of evidence pointing to the KLFs as having such a role. As critical regulators of the vascular inflammatory response in multiple tissue types, future investigations of the KLFs utilizing whole transcriptome approaches will provide valuable information regarding the breadth of KLF influence as well as potential interactions among them; these promise to be complex, as the shared consensus sequence 5′-C(A/T)CCC-3′ is prevalent throughout the genome. Additionally, the KLFs may represent attractive targets for therapeutic intervention; this will require further exploration, as the targeting of zinc-finger transcription factors remains non-trivial. Ultimately, mechanistic and therapeutic insights in KLF biology will advance our understanding of the complex signaling networks at play during vascular inflammation.

## Author Contributions

DS, LF, PH, and MJ contributed conception of the manuscript. DS wrote the first draft of the manuscript. DS, LF, PH, and MJ contributed to manuscript revision. All authors read and approved submitted version.

## Conflict of Interest Statement

The authors declare that the research was conducted in the absence of any commercial or financial relationships that could be construed as a potential conflict of interest.
